# Synovial Fluid Analysis in Melioidosis: Experiences from the Darwin Prospective Melioidosis Study

**DOI:** 10.3390/pathogens14111120

**Published:** 2025-11-03

**Authors:** Stuart Campbell, Tze I. Lee, Robert W. Baird, Ella M. Meumann, Bart J. Currie

**Affiliations:** 1 Infectious Diseases Department, Royal Darwin Hospital, Darwin, NT 0810, Australia; 2 Microbiology Department, Territory Pathology, Darwin, NT 0810, Australia; 3 Menzies School of Health Research, Charles Darwin University, Darwin, NT 0810, Australia

**Keywords:** melioidosis, septic arthritis, *Burkholderia pseudomallei*, bone and joint infection

## Abstract

Melioidosis is a multisystem disease caused by the sapronotic soil bacterium *Burkholderia pseudomallei*. Septic arthritis (SA) can occur as either a primary or secondary focus and requires surgical management with prolonged antimicrobial treatment. We used the Darwin Prospective Melioidosis Study to identify patients with melioidosis and SA, filtered by culture confirmation of *B. pseudomallei*, and subsequently collected synovial fluid analysis, laboratory, clinical, and risk factor data. We identified 68 patients in total with a label of SA, of which 46 patients supplied 69 synovial fluid samples which were culture-positive. These most commonly came from the knee (61%) and ankle (16%), though half (54%) of the specimens were clotted and unable to undergo cell count. We found a median white cell count (WCC) of 63,000 × 10^6^ cells/L in *B. pseudomallei* culture-positive samples. There was a numerical, but non-significant, difference in median synovial fluid WCC when stratified by preceding antimicrobial use (90,000 × 10^6^ cells/L prior versus 27,800 × 10^6^ cells/L in samples taken post antimicrobial initiation; *p* = 0.053). One sample was *B. pseudomallei* culture-positive 32 days following antimicrobial initiation. The presence/absence of contiguous osteomyelitis did not alter synovial fluid white cell counts. These findings suggest that in cases of suspected melioidosis SA, it is not necessary to withhold empirical antimicrobial therapy while awaiting joint aspiration. Further research is needed to define the role of non-culture-based diagnostics in suspected melioidosis SA.

## 1. Introduction

Melioidosis is a multisystem disease caused by the sapronotic soil and water environmental Gram-negative bacterium *Burkholderia pseudomallei* [1]. The disease is endemic throughout Southeast Asia and northern Australia, though cases are increasingly reported outside of these classical geographical boundaries [2,3]. Severe disease is common, with case fatality rates of between ~10 and ~35% (in high and low resource settings, respectively [4,5]).

The most common presenting clinical syndrome is pneumonia; however, many other manifestations are described [4]. A minority (~5%) of cases have septic arthritis (SA), with or without concurrent osteomyelitis (OM [4,6,7]). Accurate diagnosis of this syndrome can be challenging due to the clinical complexity of these cases with often multifocal intra- and extra-articular loci of infection. Further, our clinical experience is notable for late (days post initiation of antimicrobials) manifestations of intra-articular disease after an initial presentation with another syndrome. These osteoarticular manifestations can be challenging to manage; they typically require more invasive and aggressive surgical source control and longer durations of antimicrobial therapy than disease limited to the soft tissues [6,7,8,9].

A previous case series has described some elements of melioidosis versus non-melioidosis SA in Thailand and found increased mortality associated with melioidosis SA but similar synovial fluid analysis compared to other bacterial pathogens; the study did not report on any specific melioidosis subgroups [10]. Given the complex interplay between *B. pseudomallei* and the immune system, and our clinical experience of joint findings on clinical examination waxing and waning through the course of a patient’s illness, we considered whether synovial fluid cell count or makeup may be distinct to that of other pyogenic joint infections [11].

## 2. Methods

### 2.1. Patient Cohort and Data Collection

The Darwin Prospective Melioidosis Study (DPMS) prospectively collects clinical syndromes (including primary and secondary infectious foci), demographics, chronicity of infection, clinical risk factors and exposure history for all patients with melioidosis managed at the Royal Darwin Hospital, Northern Territory, Australia. Specific methodology and definitions of these data can be seen in the supplementary material of Reference [4]. We screened this database for all patients with a label of SA from 1 October 1999 until 1 May 2025, and then further examined those patients with growth of *B. pseudomallei* from an intra-articular clinical specimen, i.e., culture-confirmed melioidosis SA. In addition, we leveraged a previously collected osteoarticular melioidosis dataset which had documented treatment and specific site of infection data [6]. Some patients were diagnosed with melioidosis after the OAM dataset was completed, and so a proportion of data was collected de novo.

In addition to data previously collected, we interrogated the laboratory information system to identify the joints sampled, white cell count (WCC) and differential, culture result, molecular tests performed, and presence/absence of uric acid and calcium pyrophosphate dihydrate (CPPD; pseudogout) crystals in joint fluid specimens from patients included in the DPMS. Sample collection pre- or post-melioidosis-effective antimicrobial (ceftazidime or meropenem) exposure, the presence of contiguous osteomyelitis (defined by any of radiological, intraoperative, or microbiological methods), and the method (aspiration vs. operative) of sampling were also recorded. Samples were considered pre-antibiotics if they were collected before or on the same day as melioidosis-effective intravenous antibiotics (either meropenem or ceftazidime) were initiated.

### 2.2. Laboratory Measurements

Local practice in the collection of joint fluid consists of sample collection into a sterile container without an anticoagulant. Occasionally samples are also placed in ethylenediaminetetraacetic acid (EDTA) or lithium heparin tubes to reduce clotting; this allows for an accurate cell count but, due to lack of sterility, cannot be used for culture. Some labs, but not ours, use hyaluronidase to dissolve clots. When only small sample volumes are available, culture is prioritised. Biochemistry is only performed upon specific request. Cell counts are performed as soon as possible after collection, but in some instances, overnight collection is delayed until the following morning.

### 2.3. Statistical Analysis and Figure Generation

Statistical analysis was performed using StataBE (v17.0; StataCorp, College Station, TX, USA). Comparisons on fluid WCC (without log transformation) were performed using the Wilcoxon rank-sum test for binary variables and an equality of populations (Kruskal–Wallis) test for categorical variables. The relationships between categorical variables were assessed with contingency tables and the Chi^2^ test. Figures were created using Matplotlib (v3.10.3) and Seaborn (v0.13). Some figures were plotted on a log_10_ y-axis for visualisation purposes but analysed without transformation.

### 2.4. Ethics Statement

This study was approved by the Human Research Ethics Committee of the Northern Territory Department of Health and Menzies School of Health Research (approval 02/38).

## 3. Results

**Figure 1:**
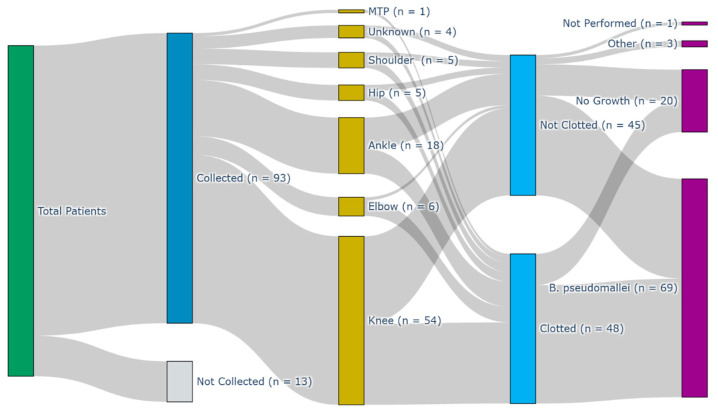
Sankey diagram of sample collection flows through the variables site of collection, whether the sample was clotted, and the end culture result. MTP—metatarsal phalangeal joint.

We identified 68 patients with a label of melioidosis SA in the DPMS dataset within our timeframe, from whom 93 joint fluid samples were collected, and of which 69 samples (from 46 individual patients) were culture-confirmed for *B. pseudomallei* (Figure 1). Thirteen patients with suspected melioidosis SA did not have samples collected (Figure 1). Sampling flows for the cohort as a whole can be seen in Figure 1. Presumed foci of SA in patients who did not undergo aspiration were typically the ankle (four foci in three patients), knee (three foci in three patients), and elbow or small joints of the foot (three foci each in three separate patients).

In the culture-confirmed cohort, mean age was 47 years, and the majority of samples were collected from the knee 61% (42/69), followed by the ankle (16%, 11/69; Figure 1). Samples in the culture-confirmed cohort were typically acquired via aspiration (58%; 40/69), with a single joint sampled in 89% (41/46), two in 7% (3/46), and three in 4% (2/46) of patients. The same joint was sampled two or more times in 46% (21/46) of patients.

Over half (54%; 37/69) of the culture-confirmed samples collected were clotted and thus unable to have cell counts performed; this proportion was similar to samples from which *B. pseudomallei* was not cultured (48%; 11/23; one sample was not cultured). There was no specific association between collection site and sample clotting (*p* = 0.524). For the 46% (32/69) culture-confirmed samples where cell counts were able to be performed, the median WCC was 63,700 × 10^6^ cells/L (interquartile range [IQR]: 17,700–132,600 × 10^6^ cells/L), with a median neutrophil proportion of 85% (IQR: 78–94). WCCs in culture-confirmed samples were significantly higher than in the 52% (12/23) of samples from the non-culture-confirmed group (median 1040 × 10^6^ cells/L; IQR: 230–3600 × 10^6^ cells/L; *p* < 0.001).

**Figure 2:**
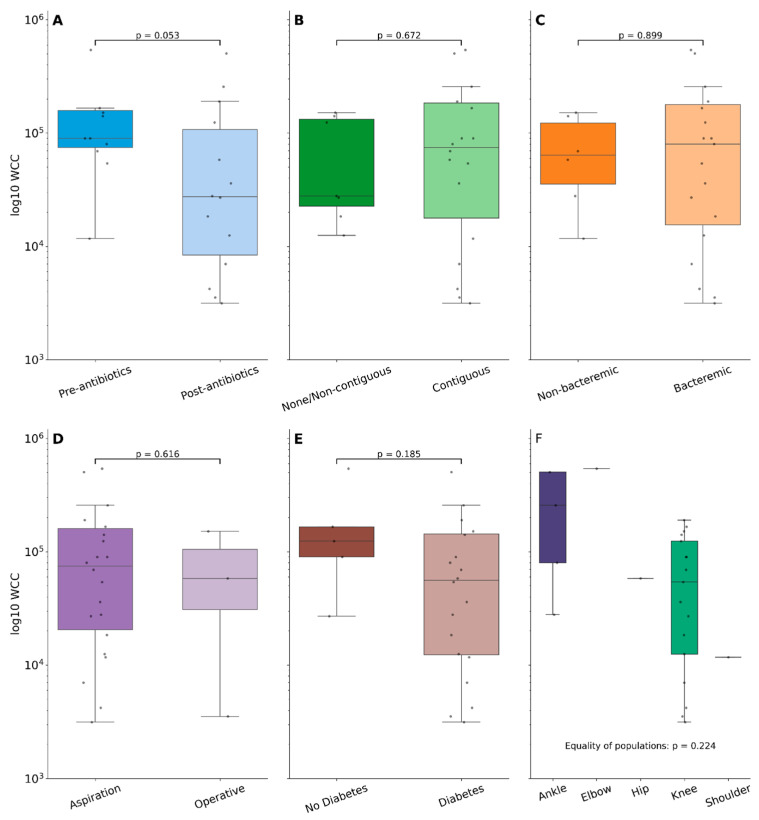
Boxplots showing log_10_ white cell count (WCC) between samples (**A**) taken pre- or post-antibiotics; (**B**) from joints with/without contiguous osteomyelitis; (**C**) from patients with/without concomitant bacteremia; (**D**) obtained via aspiration or operative methods; (**E**) stratified by diabetes status; or (**F**) stratified by the joint a sample was collected from. Groups were compared with the rank-sum test and the relevant *p*-value displayed in each subgraph.

**Figure 3:**
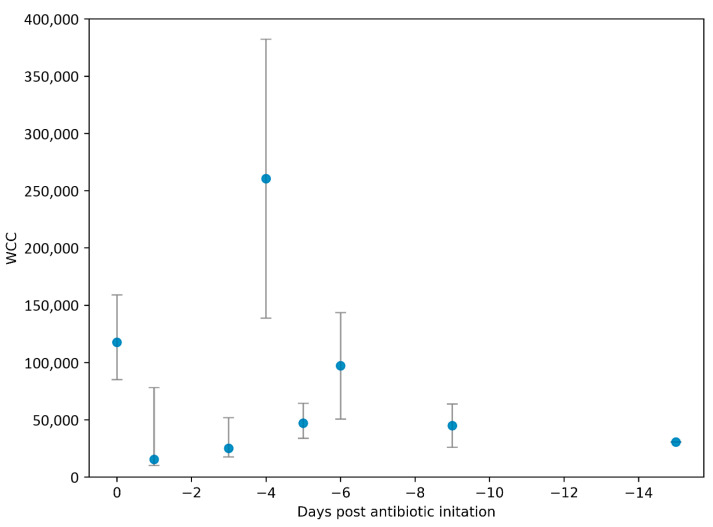
Synovial white cell counts (WCCs) stratified by the number of days post-antibiotic initiation that samples were collected. Data is displayed as median WCC bracketed by interquartile ranges.

Samples were taken prior to receipt of antibiotic therapy in 32% (22/69), with a median number of days pre-antibiotics of 1 (IQR: 0–2 days). Samples taken post-antimicrobial initiation were collected a median of 4 days post (IQR: 1–7 days), and one sample cultured *B. pseudomallei* 32 days following initiation of melioidosis therapy with ceftazidime. The WCC was numerically higher in the pre-antibiotic versus post-antibiotic sampling group (90,000 [IQR: 69,200–151,600] × 10^6^ cells/L versus 27,800 [IQR: 12,510–82,800] × 10^6^ cells/L) but did not reach statistical significance (*p* = 0.053; Figure 2A and Figure 3). Neutrophil proportions were also similar between these groups (*p* = 0.767). Subgroup analysis of the effect on fluid WCCs following treatment with antibiotics found no difference between meropenem and ceftazidime (*p* = 0.564). Furthermore, no other clinical risk factors (diabetes, bacteraemia) were associated with differences in fluid WCCs. The presence/absence of contiguous osteomyelitis did not alter synovial fluid white cell counts, and neither did the method or site of collection (Figure 2B–E).

The most common primary syndrome on admission was septic arthritis in 52% (24/46) of patients, followed by pneumonia in 37% (17/46) of patients. CPPD crystals were identified in 3% (2/69) of the samples (in samples from different patients; both samples were also culture-confirmed for *B. pseudomallei*). There were no cases with concomitant gout, as identified by the presence of uric acid crystals, in the culture-confirmed cohort, but there were two cases identified in the non-culture-confirmed cohort.

## 4. Discussion

In this study of patients with culture-confirmed melioidosis SA, we found a median WCC of 63,700 × 10^6^ cells/L when confined to samples with a positive culture of *B. pseudomallei* from the joint fluid. There exist some general guidelines for WCC thresholds in the diagnosis of septic arthritis [12,13]. These thresholds are typically set at 50,000 × 10^6^ cells/L and 100,000 × 10^6^ cells/L, the latter being highly suggestive of pyogenic SA, whilst WCCs of <25,000 × 10^6^ cells/L decrease this likelihood [12,13]. Historical reports of septic arthritis from our setting (which found *Staphylococcus aureus* to be the predominant pathogen) found similar synovial WCCs overall (79,000 × 10^6^ cells/L [14]). However, data from Thailand found a median WCC of ~30,000 × 10^6^ cells/L in culture-positive joint fluid from melioidosis patients, substantially lower than our findings [10]. These differences may be due to differences in laboratory reporting, as a number (12/77; 15%) of *B. pseudomallei*-infected joints from Teparrukkul et al. (2017 [10]) had cell counts reported only as ‘frank pus’, thus likely substantially reducing the overall median WCC.

Antibiotic therapy prior to joint fluid collection has previously been shown to reduce both the absolute number and proportion of neutrophils in native joint septic arthritis, though the predominant pathogen in that study was *S. aureus* [15]. In contrast, for *B. pseudomallei*, culture yield was not affected by preceding antimicrobials. This fits with our clinical experience of melioidosis, where patients will occasionally develop new foci of infection, including SA, even after a few weeks of high dose intravenous therapy. This is further supported by our findings of culture positivity of up to 32 days post initiation of melioidosis therapy.

**Figure 4:**
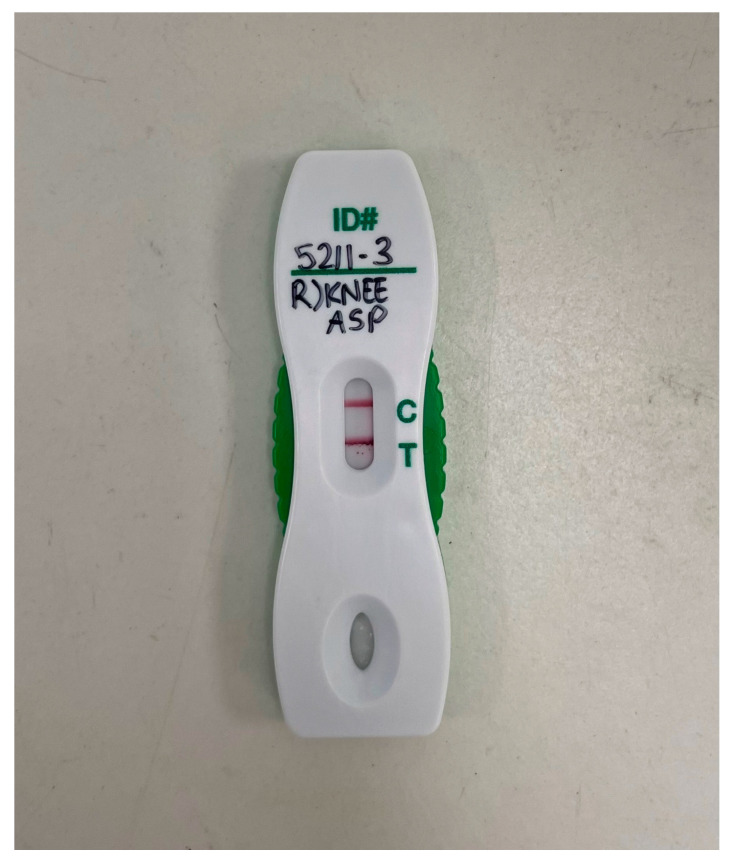
A positive melioidosis lateral flow assay (LFA) performed on specimen fluid obtained from a knee aspirate. More information on the use of this assay can be found in doi.org/10.1093/ofid/ofac149 [18].

Non-culture-based diagnostic strategies (for example, multiplexed PCR panels) are becoming more commonplace in diagnostic laboratories, and there exists an in-house real-time PCR assay for *B. pseudomallei* detection which our site employs typically for the confirmation of the identification of bacterial colonies but occasionally directly on clinical specimens [16,17]. In addition, preliminary studies of a *B. pseudomallei* capsular polysaccharide lateral flow immunoassay (LFA) found sensitivity of 46% and specificity of 100% on direct testing of joint fluid specimens (an example of positive LFA from joint fluid is shown in Figure 4 [18]).

There are several limitations to our study. Due to missing sample collection timing data, it is possible that some samples were collected many hours following antibiotic exposure, which may reduce the measured WCCs, perhaps without reducing culture yield. Furthermore, we limited assessment of samples to only culture-confirmed SA cases, a threshold that may have excluded truly infected joints. Additionally, over half of the collected samples were unable to have WCCs performed due to clotting. It is possible that these samples may be more predisposed to clot due to high inflammatory cell burden, and thus the true WCCs in melioidosis-infected joints may be higher than reported here.

In conclusion, we found that culture-confirmed melioidosis SA has similar WCC findings to those of other SA aetiologies, but culture-negative joint fluid often had a low WCC. In contrast, preceding antimicrobial therapy does not appear to affect either the WCC or culture positivity, suggesting that delaying antimicrobial therapy to improve culture yield for suspected melioidosis SA is not necessary. The role of non-culture-based diagnostic techniques in melioidosis SA such as PCR and LFA is evolving and requires ongoing research.

## Data Availability

Deidentified data can be requested from the authors.
